# Can anti-osteoporotic therapy reduce adjacent fracture in magnetic resonance imaging-proven acute osteoporotic vertebral fractures?

**DOI:** 10.1186/s12891-016-1003-1

**Published:** 2016-04-06

**Authors:** Ying-Chou Chen, Wei-Che Lin

**Affiliations:** Department of Rheumatology, Kaohsiung Chang Gung Memorial Hospital, Chang Gung University College of Medicine, 123 Ta-Pei Road, Niao-Sung District, Kaohsiung 833 Taiwan; Department of Radiology, Kaohsiung Chang Gung Memorial Hospital, Chang Gung University College of Medicine, Kaohsiung, 833 Taiwan

**Keywords:** Osteoporosis, Vertebral fracture, Adjacent fracture, Anti-osteoporotic therapy

## Abstract

**Background:**

Adjacent fracture of the cemented vertebrae result from crushed fragile trabeculae during follow-up, suggesting impaired bone marrow integrity. This study aimed to determine if anti-osteoporotic therapy can decrease the risk of adjacent fracture in patients after vertebroplasty.

**Methods:**

This retrospective study reviewed of cases of osteoporotic patients with magnetic resonance imaging (MRI)-proven acute vertebral fractures between 2001 and 2007. Osteoporotic patients were investigated as determined by pre-operative MRI with subsequent adjacent fracture of the cemented vertebrae and for the possibility of anti-osteoporotic therapy decreasing the progression of collapse after a minimum of 6 months follow-up. All associated co-morbidities were recorded, as well as the use of anti-osteoporotic drugs (i.e., bisphosphonate, raloxifen, calcitonin, and teriparatide). Cox regression analysis was also performed.

**Results:**

The 192 vertebral fractured patients who underwent vertebroplasty and anti-osteoporotic therapy had a mean age of 74.40 ± 6.41. The basic characteristics of patients with and without adjacent fracture differed in age, body mass index, rheumatoid arthritis, and use of glucocorticoids and anti-osteoporotic drugs (Table 1). Using the Kaplan-Meier curve, anti-osteoporotic therapy after vertebroplasty had a significant effect on adjacent fracture (*p* = 0.037, by log rank text). After adjusting for potential confounders, patients with anti-osteoporotic therapy still had a lower adjacent fracture rate than patients without anti-osteoporotic therapy (*p* = 0.006; HR: 2.137, 95 % CI: 1.1238–3.690). The adjacent fracture rate also increased in old age (*p* = 0.019; HR: 1.049; 95 % CI:1.008–1.039) and among smokers (*p* = 0.026; HR: 3.891; 95 % CI: 1.175–12.890).

**Conclusions:**

In this study, adjacent fracture of cemented vertebrae is inevitable after vertebroplasty but can be mitigated by anti-osteoporotic therapy to increase bone mass.

## Background

Spine fractures are common with aging. The risk of osteoporotic spine compression fracture is estimated to be 18 % in women and 11 % in men [1]. Symptomatic spine fractures increase mortality by up to 15 % [2] and some become disabled by severe pain and lasts for 2–3 months.

Acute vertebral fractures with persistent pain are frequently managed with vertebroplasty [3]. It had been widely used in recent decade. Although it had favorable clinical outcomes, few studies on mortality among patients with vertebroplasty were reported [4, 5]. Despite its safe and efficient, there are still some risks, including the development of new adjacent fractures at the non-treated vertebrae [6].

Anti-osteoporotic therapy is reported to increase bone mineral density. Patients who received anti-osteoporotic treatment reduced incidences of vertebral fractures [7]. If treating osteoporosis have benefit on adjacent fracture after vertebroplasty, it will had important implications in skeletal health care. So this study investigate whether osteoporosis treatment can affect adjacent fracture rates in patients after vertebroplasty procedure.

## Methods

The retrospective study reviewed osteoporosis patients with acute vertebral fractures which were proven by magnetic resonance imaging (MRI) and defined as low signal intensity (SI) on T1 and enhanced SI at T2-weighted of the injured vertebral body [8]. All of the patients were treated with vertebroplasty were performed in all of the patients. Chang Gang Memorial Hospital’s institutional review board reviewed and approved the study protocol, which was conducted in Good Clinical Practice Guidelines. In accordance with local government's law, no additional informed consent was required. All information was de-identified before data analysis.

Only those treated with for a single vertebral fracture were enrolled. Patients previously using anti-osteoporotic drugs were also been excluded. The medical records were reviewed and the new fractures were evaluated from the imaging follow-up.

Pre- and post-vertebroplasty radiographs and those taken more than 6 months after the procedure were obtained. Those without available radiographs were excluded from the study.

Standard methods was used to measure the height of the anterior border of the collapsed vertebral body [9]. The anterior vertebral height (AVH) was measured Differences of AVH within 1 mm were considered unchanged [10] and to avoid biases from technical factors or inappropriate measurement.

All of the study patients were recorded with, age, sex, body mass index (BMI, kg/m^2^), and all co-morbidities such as diabetes, hypertension, and liver and renal disease. The use of anti-osteoporotic drugs (i.e., alendronate, raloxifen, calcitonin, and teriparatide) were reviewed. The duration of osteoporotic therapy was followed by local government's policy with teriparatide (18 months), bisophosphonate and raloxifen, calcitonin (long term used).

### Statistical analysis

Statistical analysis was performed using the SPSS software, version 21.0 (SPSS, Chicago, IL, USA). Patient characteristics were reported as simple descriptive statistics (i.e., mean ± standard deviation [SD]). Different groups of anti-osteoporotic agents were compared using Kaplan-Meyer analysis with the log rank test. Independent *t*-test was compared for independent means, while relationships between categorical variables were evaluated by the Chi-square test. Cox regression test was used for potential confounders.

## Results

**Fig. 1:**
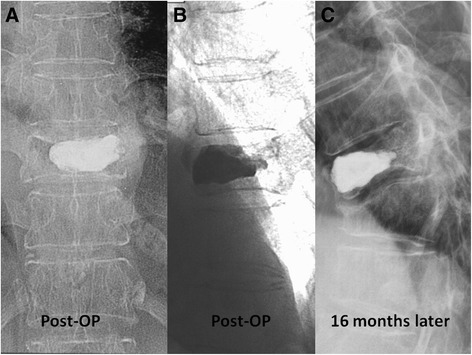
A comparison of adjacent fracture for those with (*blue line*) and without (*green line*) anti-osteoporotic therapy, by Kaplan-Meier analysis

Of 192 patients enrolled in this study, 86 (44.8 %) used alendroante, 38 (19.8 %) used raloxifen, 18 (9.4 %) used calcitonin, and 12 (6.3 %) used teriparatide. All were grade 3 by semi-quantitative grading for vertebral fracture and had a T score less than −2.5 by bone densitometry. Their mean age was 74.40 ± 6.41 years. 84 patients had adjacent fracture (Fig 1). The basic characteristic of patients with and without adjacent fracture differed in age, BMI, rheumatoid arthritis, and glucocorticoid and anti-osteoporotic therapy use (Table 1).

**Table 1:** Characteristics of the study patients with and without adjacent fracture

Variables	Adjacent fracture (*n* = 84)	No Adjacent fracture (*n* = 108)	*p* value
Age (years)	75.83 ± 6.28	73.28 ± 6.31	0.006
Body mass index (kg/m^2^)	21.68 ± 4.11	24.36 ± 4.63	0.001
Sex (% Female)	68 (81.0 %)	98 (90.7 %)	0.057
Spine fracture (number)	1.18 ± 1.33	1.89 ± 1.87	0.965
Smoking	8 (9.5)	2 (1.9)	0.023
Alcohol consumption	0 (0)	2 (1.9)	0.505
Rheumatoid arthritis	10 (11.9)	2 (1.9)	0.006
Diabetes mellitus (%)	18 (21.4)	28 (25.9)	0.5
Hypertension	44 (52.4)	48 (44.4)	0.309
Diseases			
Cardiovascular	0 (0)	2 (1.9)	0.505
Pulmonary	6 (7.1)	2 (1.9)	0.141
Liver	2 (2.4)	6 (5.6)	0.469
Glucorcorticoid use	18 (21.4)	10 (9.3)	0.023
Anti-osteoporotic drugs use	58 (69)	89 (82.4)	0.031

**Fig. 2:**
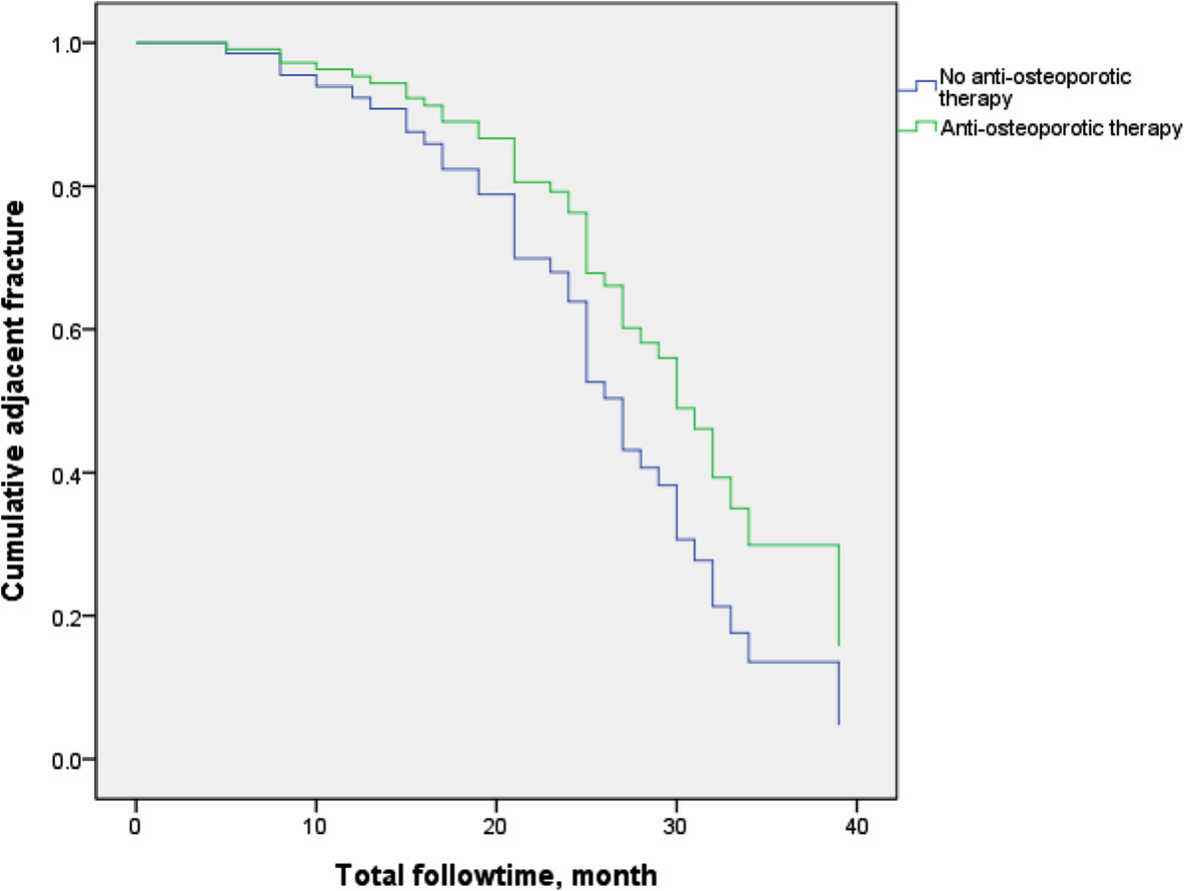
Vertebroplasty was performed for 10th thoracic vertebra (**a**, **b**). **c** Patient developed a new adjacent compression fracture of 11th thoracic vertebra 16 months later

By the Kaplan-Meier curve analysis, anti-osteoporotic treatment after vertebroplasty had a significant benefit on adjacent fracture (*p* = 0.037, by log rank test) (Fig. 2). After adjusting for confounding factors such as smoking, alcohol consumption, hypertension, diabetes, cardiovascular, pulmonary, and liver diseases, and glucocorticoid use, those with anti-osteoporotic treatment still had a lower adjacent fracture rate than those who did not receive anti-osteoporotic therapy (*p* = 0.006; HR: 2.137; 95 % CI: 1.1238–3.690). The adjacent fracture rate also increased in old age (*p* = 0.019; HR: 1.049; 95 % CI: 1.008–1.039) and among smokers (*p* = 0.026; HR: 3.891; 95 % CI:1.175–12.890) (Table 2).

**Table 2:** Multivariable Cox regression analysis of the hazard ratios for adjacent fracture

Variables	Regression coefficient	SE	Wald	*p* value	HR (95 % CI)
Age	0.048	0.021	5.471	0.019	1.049 (1.008–1.093)
Body mass index (kg/m2)	−0.055	0.036	2.344	0.126	0.946 (0.882–1.016)
Sex	−0.595	0.515	1.335	0.248	0.551 (0.201–1.513)
Smoking	1.359	0.611	4.943	0.026	3.891 (1.175–12.890)
Alcohol consumption	−13.295	383.923	0.001	0.972	0.00 (0.000)
Rheumatoid arthritis	1.238	0.634	3.817	0.051	3.448 (0.996–11.938)
Diabetes mellitus	0.262	0.300	0.762	0.383	1.298 (0.722–2.337)
Hypertension	−0.334	0.255	1.709	0.191	0.716 (0.434–1.181)
Diseases					
Cardiovascular	−12.071	442.926	0.001	0.978	0.000 (0.000)
Pulmonary	−0.159	0.552	0.082	0.774	0.853 (0.289–2.519)
Liver	−0.175	0.735	0.057	0.811	0.839 (0.199–3.547)
Glucocorticoid use	−0.304	0.442	0.472	0.492	0.737 (0.310–1.756)
Anti-osteoporotic therapy	0.759	0.279	7.426	0.006	2.137 (1.238–3.690)

*Abbreviations*: *HR* hazard ratio; *SE* standard error

Evaluating the relationship between anti-osteoporotic drug use and adjacent fracture, the use of alendronate was associated with a significant reduction in adjacent fracture (*p* = 0.011), while raloxifen, calcitoninc, and teriparatide did not decrease adjacent fracture rate (Table 3).

**Table 3:** Drugs associated with decreasing risk of adjacent fracture

Drugs	*p* value
Raloxifen	0.304
calcitonin	0.898
Teriparatide	0.878
Fosamax	0.011

## Discussion

Osteoporotic compression fractures increase the risk of new vertebral compression fracture even without percutaneous vertebroplasty [11]. Since vertebral compression fracture can lead to a collapse of adjacent vertebral bodies, it usually provokes a cascade of subsequent fractures.

Fracture of the adjacent vertebrae is a unique complication associated with vertebroplasty. The effects of vertebroplasty will increase strength of the stabilized vertebral bodies be greatest at vertebral levels near the treated vertebral body and augmented spinal segment have shown increased nucleus pulposus pressure, and lead to deformation of the adjacent endplate [12, 13], followed by decreasing segmental strength [14, 15]. Some studies report that adjacent vertebral fracture occurs sooner than non-adjacent fractures [16], and majority of cases occurring within 30 days of surgery [17]. On the other hand, other studies refute these findings and find that adjacent untreated vertebral bodies do not undergo immediate changes after vertebroplasty and that intervention did not result in adjacent vertebral fractures [18, 19].

Adjacent vertebral fractures can be prevented by correctly recognizing and performing kyphoplasty in all fractured vertebrae identified by pre-operative MRI. Furthermore, the reported inevitable side effects of long-term analgesic medication in patients with chronic pain can be avoided [20, 21] through anesthesia and more radiation.

Anti-osteoporosis includes selective estrogen receptor modulators, bisphosphonates, and parathyroid hormone analogs. Among them, bisphosphonates are the compounds most commonly used drugs, which increase bone mass in osteoporotic patients [3, 8, 15]. In a study, alendronate group had less mean loss of vertebral height after 3 years of treatment than the placebo group [15].

In this study, anti-osteoporotic therapy significantly reduces adjacent fracture. Alendronate is the main drug identified to improve adjacent fracture. Smoking and old age increases adjacent fracture so such patients warrant aggressive treatment.

This study has several limitations. First, the sample size is small. futhermore, because of the retrospective design, this study did not include datas, such as the use of calcium and vitamin D supplements. Nonetheless, in this cohort, as much data as possible was collected. Besides this study also included only fragility fractures in patients older than 50 years and exclude a secondary etiology such as cancer or pyogenic infection by MRI scans. Thus, the patients’ fractures were due to osteoporosis.

## Conclusions

Vertebroplasty can stabilize an fractured vertebra, but collapse of the cemented vertebrae can occur rapidly. In this study, we found adjacent fracture of a cemented vertebra is inevitable after vertebroplasty. Increase bone mass by anti-osteoporotic therapy after vertebroplasty may prevent the further collapse of the cemented vertebra.
